# Clinical Usefulness of the Platelet-to Lymphocyte Ratio in Patients with Angiosarcoma of the Face and Scalp

**DOI:** 10.3390/ijms18112402

**Published:** 2017-11-13

**Authors:** Gen Suzuki, Hideya Yamazaki, Norihiro Aibe, Koji Masui, Naomi Sasaki, Daisuke Shimizu, Takuya Kimoto, Jun Asai, Makoto Wada, Satoshi Komori, Norito Katoh, Kei Yamada

**Affiliations:** 1 Department of Radiology, Kyoto Prefectural University Graduate School of Medical Science, 465 Kajiicho Kawaramachi Hirokoji, Kamigyo-ku, Kyoto 602-8566, Japan; yamahi@koto.kpu-m.ac.jp (H.Y.); a-ib-n24@koto.kpu-m.ac.jp (N.A.); mc0515kj@koto.kpu-m.ac.jp (K.M.); nao-hayas@koto.kpu-m.ac.jp (N.S.); dshimizu@koto.kpu-m.ac.jp (D.S.); t-kimoto@koto.kpu-m.ac.jp (T.K.); kyamada@koto.kpu-m.ac.jp (K.Y.); 2 Department of Dermatology, Kyoto Prefectural University Graduate School of Medical Science, 465 Kajiicho Kawaramachi Hirokoji, Kamigyo-ku, Kyoto 602-8566, Japan; jasai@koto.kpu-m.ac.jp (J.A.); wada-102@koto.kpu-m.ac.jp (M.W.); f76ers@koto.kpu-m.ac.jp (S.K.); nkatoh@koto.kpu-m.ac.jp (N.K.)

**Keywords:** angiosarcoma, prognosis, distant metastasis, neutrophil-to-lymphocyte ratio, platelet-to-lymphocyte ratio, lymphocyte-to-monocyte ratio

## Abstract

Angiosarcoma of the face and scalp (ASFS) is an extremely aggressive tumor that frequently metastasizes, often leading to death. The neutrophil-to-lymphocyte ratio (NLR), platelet-to-lymphocyte ratio (PLR), and lymphocyte-to-monocyte ratio (LMR) are inflammatory markers that predict outcome of various cancers. We aimed to examine the relationship between pretreatment inflammatory markers and ASFS outcome. We included 17 patients with ASFS and a control group of 56 age- and gender-matched healthy individuals. Total white blood counts, neutrophil, lymphocyte, monocyte, and platelet counts were recorded; NLR, PLR, and LMR were calculated. Kaplan–Meier curves were used to calculate overall survival (OS) and distant metastasis-free survival (DMFS). Optimal cut-off values for each inflammatory marker were calculated using receiver operating curve analysis. Median follow-up was 22 months (range, 6–75). There was a statistically significant difference in absolute neutrophil counts and NLR between patient and control groups. Two-year OS and DMFS rates were 41% and 35%, respectively. In patients with tumors < 10 cm, PLR was highly correlated with DMFS, with the 2-year DMFS for those with a high PLR being 50% compared with 100% for those with a low PLR (*p* = 0.06). This study suggests that PLR is superior to NLR and LMR, and is a clinically useful marker in patients with ASFS with small tumors.

## 1. Introduction

Angiosarcoma is a rare malignant tumor, accounting for approximately 2% of all soft tissue sarcomas [1]. It most commonly involves the skin and subcutis, particularly the face and scalp of elderly people [2]. Angiosarcoma of the face and scalp (ASFS) is an extremely aggressive tumor, with not only a high rate of local recurrence but also a marked tendency for early metastatic spread to other organs. Previous reports have emphasized the poor prognosis of this disease, with reported 5-year survival rates of 11.1–53.8% [3]. Staging of angiosarcoma, which allows for prognostication, is based on the American Joint Committee on Cancer’s tumor, node, and metastasis staging system used for soft tissue sarcomas. However, as angiosarcoma represents a small fraction of all sarcomas, it may not be well-represented with this staging system that comprises a diversity of histologic types. Several investigators have suggested that ASFS forms a particular subgroup of sarcomas because of its extremely poor prognosis [4,5,6,7]. Therefore, it is necessary to investigate prognostic factors specific for ASFS. Although effective treatment strategies for the primary lesion are yet to be elucidated, surgery combined with chemotherapy and/or radiotherapy is considered the optimal approach to eradicating the primary tumor [3]. However, wide surgical margins are not always feasible in ASFS because of anatomic and cosmetic limitations. Therefore, radiotherapy is often used as an alternative treatment for the primary lesion [4,5,8,9]. Even with treatment, however, the majority of patients will ultimately have distant metastases, especially in the lungs. Enlarged cystic metastases arising in the peripheral lung fields may rupture, leading to repeated pneumothorax and hemothorax, which is often the cause of death [10,11].

In a recent meta-analysis by Shin et al., age, size, tumor site, surgical margin status, and surgery were all factors associated with poor prognosis [3]. While some of these factors may be useful in estimating prognosis, some are only determined after local treatment. Several tumor biomarkers, such as platelet-derived growth factor receptor-β and vascular endothelial growth factor receptor-2, may be used individually or in combination in estimate prognosis [12]. However, these biomarkers are not used in routine clinical practice due to their high cost, lack of standardization, and limited availability. Therefore, it is necessary to identify a valid, cost-effective, and reliable prognostic predictor that is available before treatment. In recent years, pretreatment subsets of peripheral blood cells, including lymphocytes, neutrophils, monocytes, and platelets, have been found to be associated with the prognosis of various cancers [13,14,15,16,17]. Pretreatment neutrophil-to-lymphocyte ratio (NLR), platelet-to-lymphocyte ratio (PLR), and lymphocyte-to-monocyte ratio (LMR) can be regarded as 3 representative systemic inflammatory markers. Several studies of such markers in soft tissue sarcoma have been reported, but there are few reports of direct comparisons of NLR, PLR, and LMR [18,19,20]. In addition, there has been no research of their relationship with angiosarcoma alone. The purposes of this study were to clarify the relationship between pretreatment inflammatory markers in the peripheral blood and the outcome of patients with angiosarcoma and to evaluate which of these inflammatory markers would be most the useful predictors of distant metastasis-free survival (DMFS).

## 2. Results

### 2.1. Basic Characteristics of the Study Sample

Our study sample comprised 17 patients with ASFS and 56 normal healthy controls. Patients had a median age of 76 (range: 57–84) years, with a gender distribution of 11 males (64.7%) and 6 females (35.3%). The median age in the control group was 75 (range: 59–88) years with a gender distribution of 38 males (67.9%) and 18 females (32.1%). No statistically significant differences were observed in age and gender between the patient and control groups (*p* = 0.82 and *p* = 1.00, respectively). Comparisons among the patient and control groups with respect to laboratory findings are shown in Table 1. We found that the patients with ASFS had higher pretreatment neutrophil counts than healthy controls (*p* = 0.013). NLR was 2.63 ± 0.94 in the patient group and 1.93 ± 0.81 in the control group. The NLR value of the patients was significantly higher than that of the control group (*p* = 0.0063).

### 2.2. Patient Characteristics

Seventeen patients treated at our hospital were included in this study. The distribution of primary lesions was as follows: 15 on the scalp, 1 on the face, and 1 on the ear. A total of 11 men and 6 women with ages ranging from 57 to 84 years (median, 76) were included. The Eastern Cooperative Oncology Group performance status was 0–1 in all cases. The maximal primary tumor size on clinical assessment ranged from 1 to 12 (median, 4) cm. The number of tumors was defined as the number of purpuric lesions and was classified for each patient as solitary or multiple.

### 2.3. Optimal Cut-Off Values for NLR, PLR, and LMR

Receiver operating curves (ROCs) for DMFS were plotted to verify the optimal cut-off values for NLR, PLR, and LMR, resulting in values of 2.05 for NLR, 124.4 for PLR, and 3.24 for LMR. The highest specificity and sensitivity were 0.5 and 0.818 for NLR, 0.722 and 0.833 for PLR, and 0.5 and 0.909 for LMR. The areas under the curve (AUCs) were 0.682 for NLR, 0.803 for PLR, and 0.606 for LMR.

### 2.4. Recurrence Patterns

Recurrence was observed in 12 of the 17 patients (71%), including local recurrence alone in 3, distant organ metastases alone in 1, and both local and distant disease in 8. Local recurrence therefore occurred in 11 of 17 patients (64%) and distant metastases in 9 (53%). Local recurrence in all cases developed within the irradiated field. Overall, the lungs were the most frequent site of distant metastases (8 of 9 patients).

### 2.5. Treatment Outcome

**Figure 1:**
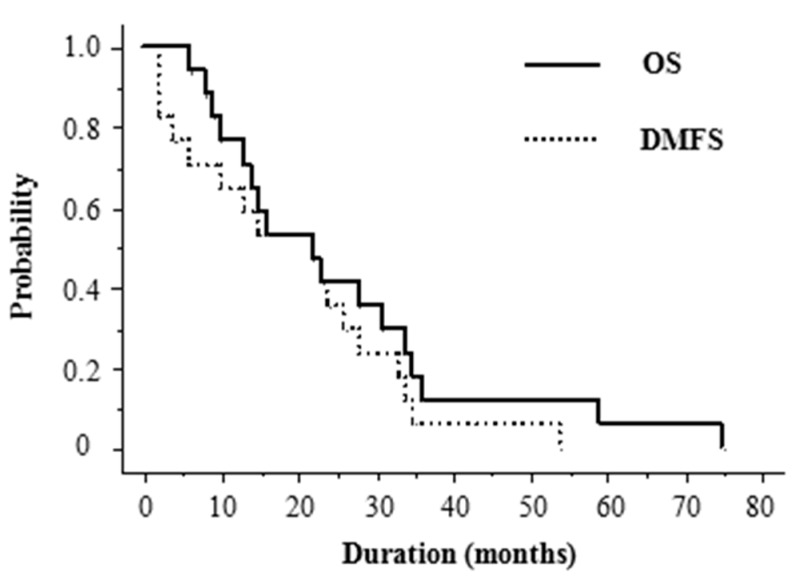
Kaplan–Meier curves for overall survival (OS) and distant metastasis-free survival (DMFS) of patients with angiosarcoma of the face and scalp (ASFS).

**Figure 2:**
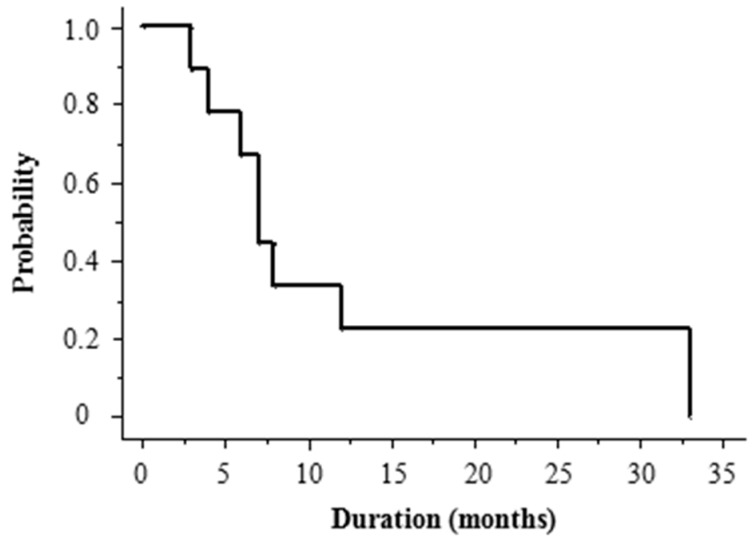
Kaplan–Meier curves for OS after distant metastases of patients with ASFS.

**Figure 3:**
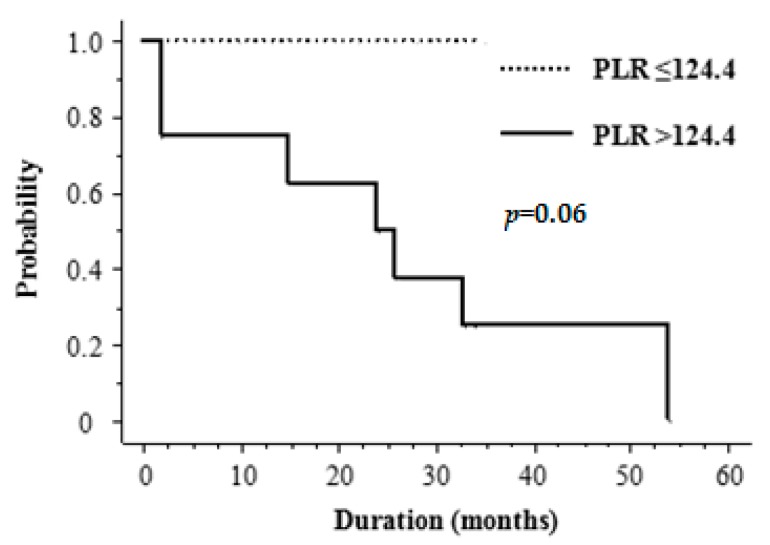
Kaplan–Meier curves for DMFS of patients with tumors <10 cm by low vs. high platelet-to-lymphocyte ratio (PLR).

The median follow-up period was 22 months (range, 6–75). At the last follow-up, 10 patients (59%) had died. Overall survival (OS) was 77% at 1 year and 41% at 2 years, with a median OS of 22 months. DMFS was 65% at 1 year and 35% at 2 years, with a median DMFS of 22 months (Figure 1). The prognosis after the development of distant metastases was dismal, with a 1 year OS of 22% and median survival of 7 months (range, 3–33 months) (Figure 2). The high frequency of distant metastases prompted us to investigate potential predictive factors for DMFS. Table 2 shows the results of univariate analysis of the various factors that might be associated with DMFS. Tumor size was significantly correlated with DMFS. The DMFS of patients with tumors <10 cm in size was significantly better than that of those with tumors ≥10 cm (*p* = 0.03). None of the inflammatory markers were significantly correlated with DMFS in the total study population. Among patients with tumors <10 cm (Table 3), a PLR of >124.4 seemed to be an important prognostic factor for DMFS, but this was not of statistical significance (*p* = 0.06; Figure 3). Table 4 summarizes patients’ characteristics and laboratory findings in all 17 patients.

## 3. Discussion

Owing to the rarity of angiosarcoma, only a few reports have evaluated prognostic factors related to distant metastases. The purpose of this study of a case series from a single institution was to examine the prognostic factors for DMFS in ASFS and to investigate the clinical significance of several inflammatory markers. The results indicated that pretreatment PLR may correlate with DMFS in patients with tumors < 10 cm in size.

Inflammation is a hallmark of cancer [21]. There is often a complex host–tumor relationship, with most tumors having inflammatory cells and mediators present in their microenvironment [22,23]. Neutrophils are at the front line of the defense system and are thought to produce several cytokines and angiogenic factors that participate in different steps in tumor development [24]. Lymphocytes are known to act as the host defense against tumor cells. Previous studies have demonstrated an association between a low peripheral lymphocyte count and short survival in different types of cancer [25,26]. Platelets are part of the inflammatory response as well, and thrombocytosis is common in patients with solid tumors [27,28]. Platelets are known to interact directly with tumor cells and contain factors that contribute to tumor growth, invasion, and angiogenesis [29]. They can protect tumor cells from natural killer cell-mediated lysis, thereby facilitating metastasis [30]. Correlations between increased platelet counts and shorter survival time have been reported for the majority of common cancer entities, including breast, lung, colon, esophageal, gastric, renal transitional cell, endometrial, and ovarian cancers, as well as melanoma and glioblastoma [31]. Investigators have reported that inflammatory markers such as PLR, NLR, and LMR can be used to predict recurrence and death for various cancers [13,14,15,16,17]. Regardless of the site of the cancer, a high NLR and PLR and a low LMR have been reported to be associated with increased mortality and recurrence rates. A meta-analysis assessing the association of PLR and OS in 12,754 patients with various solid tumors concluded that a high PLR is associated with a significantly worse prognosis [15].

No previous study has targeted angiosarcoma alone, although there are a few reports examining the relationship between survival and inflammatory markers in soft tissue sarcomas [18,19,20]. Choi et al. assessed multiple preoperative inflammatory serum markers, including C-reactive protein, erythrocyte sedimentation rate, and NLR, and found an association between high levels of inflammatory markers and shorter disease-specific survival [18]. On the other hand, Que et al. found on multivariate analysis that preoperative PLR but not NLR was an independent predictor of disease-free survival [19]. Interestingly, Szkandera et al. found that high preoperative LMR, but not PLR or NLR, was an independent prognostic factor for disease-free survival in patients with soft tissue sarcoma [20]. These authors studied different cancer populations, different NLR and PLR cut-off values, and patient cohorts with different median ages. In addition, studies of these inflammatory factors may be affected by potential confounding factors, including ethnicity, smoking history, performance status, and comorbidities. For example, Asians generally have lower peripheral blood neutrophil counts and higher lymphocyte counts than do Caucasians [32].

In order to inform Japanese clinical practice with regard to ASFS, we identified the ideal cut-off values by assessing ROC curves. The fact that, in the total study cohort, a high NLR and PLR and a low LMR were not necessarily associated with a worse DMFS was an unexpected finding. However, for patients with tumors smaller than 10 cm, the 2-year DMFS for those with a high PLR was 50%, compared with 100% for those with a low PLR (Figure 3). The reason for the association between tumor size and pretreatment PLR remains unclear, but it may reflect differences in the biology of extremely large primary tumors. Also patients with extremely large primary tumors are at risk for severe stress and for bacterial infections within the primary tumor, factors which may easily affect platelet and lymphocyte counts. To the best of our knowledge, this is the first study to investigate the clinical significance of inflammatory markers in ASFS. Furthermore, we demonstrated, for the first time, that patients with ASFS had significantly higher NLR than healthy controls, although NLR was not a prognostic factor in ASFS.

Several previous studies reported that chemotherapy was an effective treatment for ASFS [9,33,34]. One major limitation of our study is the heterogeneity in the therapeutic methods used, especially in terms of the chemotherapy regimens. The usage and regimen of chemotherapy were determined by physicians according to each patient’s general condition and function of organs, such as the kidney, liver, heart, and bone marrow. Distant metastases developed early after local treatment in two patients who could not be treated with chemotherapy. However, it is certainly possible that there could be a selection bias in our small retrospective study. It was not our aim and it is beyond the scope of this study to discuss the role of chemotherapy in the control of distant metastases.

In our study, PLR seemed to be an important prognostic factor for DMFS in patients with tumors < 10 cm, although it was not statistically significant. We showed PLR could be an effective marker for estimating distant metastases in two cases with a tumor size of just 1 cm. Our results showed that PLR, an inexpensive and readily available biomarker, may be useful in clinical situations for ASFS with small tumors. Further large-scale investigations are needed to establish the role of inflammatory markers with specific cut-off values in patients with ASFS.

## 4. Patients and Methods

### 4.1. Study Design and Population

A single center retrospective cohort study was conducted at the Kyoto Prefectural Medical University Hospital, Kyoto, Japan. Patients identified from medical records were included in this study when they met all of the following criteria: clinically diagnosed ASFS on the basis of biopsy and diagnostic imaging such as magnetic resonance imaging and/or positron emission tomography (PET); no distant organ metastases before initiation of radiotherapy; no history of radiotherapy; local field radiotherapy given with or without chemotherapy between January 2000 and December 2016 in the University Hospital Kyoto Prefectural University of Medicine. We retrospectively reviewed medical records of these patients. To compare the biomarker profile, 56 age- and gender-matched normal healthy individuals who came to our hospital for regular health checkup were also included in the present study. The study was approved by the Institutional Review Board of Kyoto Prefectural University of Medicine with permission code ERB-C-951 on 21 September 2017.

### 4.2. Treatment

All patients were treated with radiotherapy delivered with a 5- to 9-MV electron beam using techniques appropriate to the site of the tumor. Daily fractions of 2.0–3.0 Gy for 5 days per week were used. The median total radiation dose was 75 Gy (range, 60.0–100.0) with an optimal shrinking field technique at a dose of 50–60 Gy and a median dose per fraction of 2.0 Gy. The primary site was irradiated using extended local fields. The primary tumor and all satellite lesions with a 3- to 5-cm margin were encompassed by single or multiple matched appositional electron fields. Surgery was performed in 4 patients (in 1 patient following and in 3 patients before radiotherapy). Fifteen patients received chemotherapy with docetaxel concurrent with radiotherapy. Docetaxel was given at 20–60 mg/m^2^ biweekly or triweekly. The same regimen was continued after radiotherapy until disease progression or unacceptable toxicity occurred. Adjuvant immunotherapy using recombinant interleukin-2 was given before and after radiotherapy in 7 patients by intratumoral injection.

### 4.3. Hematologic Parameters Measured

We retrospectively collected the following data from patient clinical records: total white blood cell count (expressed as 10^9^ cells/L); absolute neutrophil, lymphocyte, and monocyte counts (expressed as 10^9^ cells/L); and platelet count (expressed as 10^9^ cells/L).

These values were used to calculate the NLR, PLR, and LMR.

The hematologic parameters were collected for all patients within 2 weeks before initial treatment.

### 4.4. Statistical Analysis

The age distribution of two groups was analyzed by a Mann–Whitney *U*-test. The data are presented as median (range). Categorical variables were summarized as percentages of the group total and comparisons between groups were analyzed using the chi-square test. The significance of differences of laboratory data between two groups is determined using an unpaired Student’s *t*-test. The laboratory data are presented as mean ± standard deviation (SD). Distant metastasis was defined as an apparent tumor recurrence detected by computed tomography (CT) and/or PET/CT after initial treatment. OS and DMFS were calculated according to the Kaplan–Meier method, starting from the day initial treatment began. Differences between groups were estimated using the log-rank test. Optimal cut-off values for the PLR, NLR, and LMR were calculated by analyzing the ROC and AUC. All statistical analyses were performed with EZR (Saitama Medical Center, Jichi Medical University, Saitama, Japan), a graphical user interface for R (The R Foundation for Statistical Computing, Vienna, Austraria). More precisely, it is a modified version of R commander designed to add statistical functions frequently used in biostatics [35]. All analyses used the conventional *p* < 0.05 level of significance.

## 5. Conclusions

This study suggests that PLR is superior to NLR and LMR and is a clinically useful marker in patients with ASFS with small tumors. Further studies including larger numbers of patients with longer follow-up periods are needed to determine the validity of this result.

## Figures and Tables

**Table 1:** Values for total white blood cells, neutrophils, lymphocytes, monocytes, platelet counts, neutrophil-to-lymphocyte ratios, platelet-to-lymphocyte ratios, and monocyte-to-lymphocyte ratios.

Blood Components	Patients Group (*n* = 17)	Control Group (*n* = 56)	*p* Value
Total white blood cells (×10^9^/L)	6.22 ± 1.22	5.64 ± 1.38	0.21
Absolute neutrophil count (×10^9^/L)	3.90 ± 1.06	3.16 ± 0.95	0.013
Absolute lymphocyte count (×10^9^/L)	1.58 ± 0.36	1.77 ± 0.55	0.20
Absolute monocyte count (×10^9^/L)	0.45 ± 0.14	0.47 ± 0.16	0.32
Total platelets (×10^9^/L)	201.38 ± 42.72	205.32 ± 39.55	0.49
NLR	2.63 ± 0.94	1.93 ± 0.81	0.0063
PLR	131.91 ± 36.62	125.33 ± 44.77	0.59
LMR	3.83 ± 0.99	3.81 ± 1.72	0.96

Abbreviations: NLR, neutrophil-to-lymphocyte ratio; PLR, platelet-to-lymphocyte ratio; LMR, lymphocyte-to-monocyte ratio.

**Table 2:** Univariate analysis of potential prognostic variables for distant metastasis-free survival (DMFS) of patients with angiosarcoma of the face and scalp (ASFS).

Variable	Number of Patients	DMFS	
		2-Year Rate	*p* Value
		(%)	
Age (years)			
<78	9	44	
≥78	8	25	0.2
Sex			
Male	11	36	
Female	6	33	0.2
Tumor size (cm)			
<10 cm	13	46	
≥10 cm	4	0	0.03
Number of tumors			
Solitary	6	20	
Multiple	11	42	0.1
PLR			
≤124.4	8	25	
>124.4	9	44	0.5
NLR			
≤2.05	11	57	
>2.05	6	33	0.8
LMR			
≤3.24	4	40	
>3.24	13	33	0.9

Abbreviations: DMFS: distant metastasis-free survival; NLR: neutrophil-to-lymphocyte ratio; PLR: platelet-to-lymphocyte ratio; LMR: lymphocyte-to-monocyte ratio.

**Table 3:** DMFS in patients with ASFS < 10 cm in size according to inflammatory markers.

Variable	Number of Patients	DMFS	
		2-Year Rate	*p* Value
		(%)	
PLR			
≤124.4	5	100	
>124.4	8	50	0.06
NLR			
≤2.05	5	80	
>2.05	8	55	0.7
LMR			
≤3.24	4	75	
>3.24	9	61	0.4

Abbreviations: DMFS: distant metastasis-free survival; NLR: neutrophil-to-lymphocyte ratio; PLR: platelet-to-lymphocyte ratio; LMR: lymphocyte-to-monocyte ratio.

**Table 4:** Summary of patient’s characteristics and laboratory findings.

Patient	Age (Year)	Gender	Tumor Size (cm)	Sites of Metastases	PLR	NLR	LMR
1	76	male	1	Lung and Liver	157.28	2.51	4.89
2	57	male	1	Lung	228.70	2.06	4.30
3	78	male	1.5	-	142.00	3.21	2.33
4	76	male	2.3	-	118.03	3.14	7.06
5	67	male	2.8	-	100.23	1.71	3.01
6	84	female	3	Lung	151.74	3.33	2.59
7	73	female	3	Lung	142.38	4.02	5.40
8	80	female	3.5	-	182.37	5.11	3.52
9	74	male	4	Lung	124.55	1.66	3.11
10	78	male	5.5	-	83.83	1.36	3.59
11	82	male	5.5	-	79.66	1.57	2.19
12	76	female	7	Lung	185.48	1.78	3.22
13	75	male	8	-	105.63	2.26	4.53
14	78	male	10	-	106.24	2.67	4.56
15	83	female	10	Lung	176.63	4.88	3.08
16	74	female	11	Bone	55.20	1.63	4.47
17	79	male	12	Lung	102.53	1.75	3.25

Abbreviations: DMFS: distant metastasis-free survival; NLR: neutrophil-to-lymphocyte ratio; PLR: platelet-to-lymphocyte ratio; LMR: lymphocyte-to-monocyte ratio.

## Abbreviations

ASFS	angiosarcoma of the face and scalp
NLR	neutrophil-to-lymphocyte ratio
PLR	platelet-to-lymphocyte ratio
LMR	lymphocyte-to-monocyte ratio
ASFS	angiosarcoma of the face and scalp
DMFS	distant metastasis-free survival
ROC	receiver operating curve
AUC	area under the curve
OS	overall survival
PET	positron emission tomography
CT	computed tomography

## References

[1] Fury M.G., Antonescu C.R., Van Zee K.J., Brennan M.F., Maki R.G. (2005). A 14-year retrospective review of angiosarcoma: Clinical characteristics, prognostic factors, and treatment outcomes with surgery and chemotherapy. Cancer J..

[2] Hodgkinson D.J., Soule E.H., Woods J.E. (1979). Cutaneous angiosarcomas of the head and neck. Cancer.

[3] Shin J.Y., Roh S.G., Lee N.H., Yang K.M. (2017). Predisposing factors for poor prognosis of angiosarcoma of the scalp and face: Systematic review and meta-analysis. Head Neck.

[4] Pawlik T.M., Paulino A.F., McGinn C.J., Baker L.H., Cohen D.S., Morris J.S., Rees R., Sondak V.K. (2003). Cutaneous angiosarcoma of the scalp: A multidisciplinary approach. Cancer.

[5] Sasaki R., Soejima T., Kishi K., Imajo Y., Hirota S., Kamikonya N., Murakami M., Kawabe T., Ejima Y., Matsumoto A. (2002). Angiosarcoma treated with radiotherapy: Impact of tumor type and size on outcome. Int. J. Radiat. Oncol. Biol. Phys..

[6] Abraham J.A., Hornicek F.J., Kaufman A.M., Harmon D.C., Springfield D.S., Raskin K.A., Mankin H.J., Kirsch D.G., Rosenberg A.E., Nielsen G.P. (2007). Treatment and outcome of 82 patients with angiosarcoma. Ann. Surg. Oncol..

[7] Kotilingam D., Lev D.C., Lazar A.J.F., Pollock R.E. (2006). Staging soft tissue sarcoma: Evolution and change. CA Cancer J. Clin..

[8] Suzuki G., Yamazaki H., Takenaka H., Aibe N., Masui K., Kimoto T., Tatekawa K., Nakashima A., Takenaka T., Asai J. (2016). Definitive radiation therapy for angiosarcoma of the face and scalp. Vivo.

[9] Fujisawa Y., Yoshino K., Kadono T., Miyagawa T., Nakamura Y., Fujimoto M. (2014). Chemoradiotherapy with taxane is superior to conventional surgery and radiotherapy in the management of cutaneous angiosarcoma: A multicentre, retrospective study. Br. J. Dermatol..

[10] Ito T., Uchi H., Nakahara T., Tsuji G., Oda Y., Hagihara A., Furue M. (2016). Cutaneous angiosarcoma of the head and face: A single-center analysis of treatment outcomes in 43 patients in Japan. J. Cancer Res. Clin. Oncol..

[11] Nomura M., Nakaya Y., Saito K., Miyoshi H., Kishi F., Hibino S., Saijyo T., Ito S., Nakagawa K., Nakanishi H. (1994). Hemopneumothorax secondary to multiple cavitary metastasis in angiosarcoma of the scalp. Respiration.

[12] Yonemori K., Tsuta K., Ando M., Hirakawa A., Hatanaka Y., Matsuno Y., Chuman H., Yamazaki N., Fujiwara Y., Hasegawa T. (2011). Contrasting prognostic implications of platelet-derived growth factor receptor-β and vascular endothelial growth factor receptor-2 in patients with angiosarcoma. Ann. Surg. Oncol..

[13] Roxburgh C.S.D., McMillan D.C. (2010). Role of systemic inflammatory response in predicting survival in patients with primary operable cancer. Future Oncol..

[14] Nishijima T.F., Muss H.B., Shachar S.S., Tamura K., Takamatsu Y. (2015). Prognostic value of lymphocyte-to-monocyte ratio in patients with solid tumors: A systematic review and meta-analysis. Cancer Treat. Rev..

[15] Templeton A.J., Ace O., McNamara M.G., Al-Mubarak M., Vera-Badillo F.E., Hermanns T., Eruga B., Ocana A., Tannock I.F., Amir E. (2014). Prognostic role of platelet to lymphocyte ratio in solid tumors: A systematic review and meta-analysis. Cancer Epidemiol. Biomark. Prev..

[16] Kano S., Homma A., Hatakeyama H., Mizumachi T., Sakashita T., Kakizaki T., Fukuda S. (2017). Pretreatment lymphocyte-to-monocyte ratio as an independent prognostic factor for head and neck cancer. Head Neck.

[17] Piciucchi M., Stigliano S., Archibugi L., Zerboni G., Signoretti M., Barucca V., Valente R., Fave G.D., Capurso G. (2017). The neutrophil/lymphocyte ratio at diagnosis is significantly associated with survival in metastatic pancreatic cancer patients. Int. J. Mol. Sci..

[18] Choi E.S., Kim H.S., Han I. (2014). ElevatedpPreoperative systemic inflammatory markers predict poor outcome in localized soft tissue sarcoma. Ann. Surg. Oncol..

[19] Que Y., Qiu H., Li Y., Chen Y., Xiao W., Zhou Z., Zhang X. (2015). Preoperative platelet-lymphocyte ratio is superior to neutrophil-lymphocyte ratio as a prognostic factor for soft-tissue sarcoma. BMC Cancer.

[20] Szkandera J., Gerger A., Liegl-Atzwanger B., Absenger G., Stotz M., Friesenbichler J., Trajanoski S., Stojakovic T., Eberhard K., Leithner A. (2014). The lymphocyte/monocyte ratio predicts poor clinical outcome and improves the predictive accuracy in patients with soft tissue sarcomas. Int. J. Cancer.

[21] Hanahan D., Weinberg R.A. (2011). Review hallmarks of cancer: The next generation. Cell.

[22] Mantovani A., Mantovani A., Allavena P., Allavena P., Sica A., Sica A., Balkwill F., Balkwill F. (2008). Cancer-related inflammation. Nature.

[23] Grivennikov S.I., Greten F.R., Karin M. (2011). Immunity, inflammation, and cancer. Cell.

[24] Tecchio C., Scapini P., Pizzolo G., Cassatella M.A. (2013). On the cytokines produced by human neutrophils in tumors. Semin. Cancer Biol..

[25] Mohammed Z.M., Going J.J., Edwards J., Elsberger B., Doughty J.C., McMillan D.C. (2012). The relationship between components of tumour inflammatory cell infiltrate and clinicopathological factors and survival in patients with primary operable invasive ductal breast cancer. Br. J. Cancer.

[26] Rabinowich H., Cohen R., Bruderman I., Steiner Z., Klajman A. (1987). Functional analysis of mononuclear cells infiltrating into tumors: Lysis of autologous human tumor cells by cultured infiltrating lymphocytes. Cancer Res..

[27] Wagner D.D. (2005). New links between inflammation and thrombosis. Arterioscler. Thromb. Vasc. Biol..

[28] Stone R.L., Nick A.M., McNeish I.A., Balkwill F., Han H.D., Bottsford-Miller J., Rupaimoole R., Armaiz-Pena G.N., Pecot C.V., Coward J. (2012). Paraneoplastic thrombocytosis in ovarian cancer. N. Engl. J. Med..

[29] Jain S., Harris J., Ware J. (2010). Platelets: Linking hemostasis and cancer. Arterioscler. Thromb. Vasc. Biol..

[30] Nieswandt B., Hafner M., Echtenacher B., Männel D.N. (1999). Lysis of tumor cells by natural killer cells in mice is impeded by platelets. Cancer Res..

[31] Buergy D., Wenz F., Groden C., Brockmann M.A. (2012). Tumor-platelet interaction in solid tumors. Int. J. Cancer.

[32] Bain B., Seed M., Godsland I. (1984). Normal values for peripheral blood white cell counts in women of four different ethnic origins. J. Clin. Pathol..

[33] Asmane I., Litique V., Heymann S., Marcellin L., Métivier A.C., Duclos B., Bergerat J.P., Kurtz J.E. (2008). Adriamycin, cisplatin, ifosfamide and paclitaxel combination as front-line chemotherapy for locally advanced and metastatic angiosarcoma. Analysis of three case reports and review of the literature. Anticancer Res..

[34] Skubitz K.M., Haddad P.A. (2005). Paclitaxel and pegylated-liposomal doxorubicin are both active in angiosarcoma. Cancer.

[35] Kanda Y. (2013). Investigation of the freely available easy-to-use software “EZR” for medical statistics. Bone Marrow Transplant..

